# Factors Influencing Neurosurgeons’ Decision to Retain in a Work Location: A Qualitative Study

**DOI:** 10.5539/gjhs.v7n5p333

**Published:** 2015-04-03

**Authors:** Sima Rafiei, Mohammad Arab, Arash Rashidian, Mahmood Mahmoudi, Vafa Rahimi-Movaghar

**Affiliations:** 1 Department of Management and Health Economics, School of Public Health, Tehran University of Medical Sciences, Tehran, Iran; 2 Department of Biostatistics and Epidemiology, School of Public Health, Tehran University of Medical Sciences, Tehran, Iran; 3 Sina Trauma and Surgery Research Center, Sina Hospital, Tehran University of Medical Sciences, Tehran, Iran

**Keywords:** factor, health workforce, physician, retention, rural area, work location

## Abstract

**Introduction:**

Physician retention is a serious concern to have an effective and efficient health system; the key challenge is how best to encourage and retain health providers in their work location. There have been considerable studies on factors influencing physicians’ retention but little research has been conducted in Iran. This study aims to determine the affecting factors from neurosurgeons’ viewpoint to support policy makers in proposing a sort of evidence based retention strategies.

**Methods:**

We conducted semi structured interviews with 17 neurosurgeons working in 9 provinces of Iran between September and November 2014. We included physicians remaining to work in a particular community for at least 3 years and asked them about the factors influenced their decision to retain in a work place. Data were thematically analyzed using “framework approach” for qualitative research.

**Results:**

Satisfaction with monetary incentives, availability of adequate clinical infrastructure in a community and appropriate working condition were most commonly cited factors mentioned by all participants as key reasons for retention. Furthermore elements which contributed to the quality of living condition, personal background and incentives, family convenience were emphasized by majority of them. A small number of participants mentioned opportunity for continuing learning and updating knowledge as well as supportive organizational policies as important motivators in a workplace.

**Conclusion:**

Ministry of Health and Medical Education (MOHME) should consider a multifaceted and holistic approach to improve neurosurgeons’ retention in their work location. Our findings suggests a combination of financial remuneration, establishment of adequate hospitals and clinical facilities, collaborative working environment with reasonable workload, proper living condition, family support and facilities for professional development to be employed as an effective strategy for promoting physicians’ retention.

## 1. Introduction

Policy makers world-wide aim to actively meet, in a fair and equal manner, the growing health needs of their population, especially those living in remote or underserved areas (*Increasing access to health workers in remote and rural areas through improved retention: global policy recommendations*, 2010). Adequate number of skilled and motivated health providers at the right place and the right time is vital to guarantee a well performing healthcare system capable of delivering high quality health services and improving health outcomes (S. Anand & Bärnighausen, 2007; *Improving Health Worker Performance: in Search of Promising Practices*, 2006; *Increasing access to health workers in remote and rural areas through improved retention: global policy recommendations*, 2010; Robinson, Wharrad, & Harrad, 2000; Speybroeck, Kinfu, Dal Poz, Evans, & A, 2006; Travis et al., 2004; Troy, Wyness, & Mc Auliffe, 2007; *Working together for health*, 2006; Zurn, Codjia, Sall, & Braichet, 2010). Shortage of health care providers in remote and underserved areas in almost every country is an important barrier to provide the highest achievable level of health and thus severely hampers the ability to attain national and global health goals (Dussault & Franceschini, 2006; Kirigia, Gbary, Muthuri, Nyoni, & A., 2006; Muula, Panulo, & Maseko, 2006; *Shaping the future*, 2003). Such shortages are key indications of an ineffective health care system which is to some extent the result of insufficient health care manpower production and to the greater importance is related to health care system failure to maintain workers in places where they are most needed (*Working together for health*, 2006). Longer duration of stay in a work location may be linked to increased familiarity and experience of manpower from local health needs and continuity of services in a community (J. Humphreys, Wakerman, Pashen, & Buykx, 2009; *Improving Health Worker Performance: in Search of Promising Practices*, 2006). In contrast, staff turnover will negatively affect the quality of healthcare being provided and accessibility to care. It also causes extra costs due to additional recruitment and replacements (Buchan & Seccombe, 1991; *Improving Health Worker Performance: in Search of Promising Practices*, 2006; Troy et al., 2007; Waldman, Kelly, Aurora, & al., 2004). As it seems one of the main challenges is to improve health manpower retention in work locations particularly remote and underserved areas (Buchan, 2010; *Increasing access to health workers in remote and rural areas through improved retention: global policy recommendations*, 2010). This is a global problem that almost all countries deal with (Chen, 2010; Dussault & Franceschini, 2006; Rao, Rao, Kumar, Chatterjee, & Sundararaman, 2011; *Shaping the future*, 2003). In low and middle income countries this problem is even more prominent (Chomitz, Setiadi, & Azwar, 1998; J. Humphreys & Dixon, 2004; Kirigia et al., 2006; Muula et al., 2006). In Iran, although there was a substantial increase in the number of physicians trained, still about 2000 general practitioners (GPs) and 1700 specialists are needed to serve in remote and rural areas (“Factors Influencing physician retention in underserved areas,” 2013). Currently near to 2200 specialists are working in about 270 underserved areas not being satisfied with their income and work condition. Most of the professionals do not prefer to continue serving in such remote areas and seek an opportunity to leave in a shortest time possible (“A plan for physician retention in rural and underserved areas,” 2014). High physician turnover leads to continuing shortage of physicians in rural and remote communities and significant concentration in large urban areas (S. Anand & Bärnighausen, 2004; Pong & Pitblado, 2005). In response to maldistribution, there should be an effective strategy for improving physicians’ retention in such areas (Pathman, Konrad, Dann, & al., 2004; H. K. Rabinowitz, 1993; H. K. Rabinowitz, Diamond, Gayle, Turner, & Rosenthal, 1998). A national program entitled “evolution in health system of Iran” was introduced and implemented on May 2014 to overcome obstacles in population access to health care services and increase the possibility of receiving high quality, timely and appropriate services among people (Note 1). One of the main objectives of this program is to apply equitable distribution of GPs and specialists within a country and improve their retention in work locations particularly remote and underserved areas (Note 2).

Furthermore as a result of an ageing population, sedentary life styles among Iranian people and dominant increase in the rate of road accidents need for neurosurgery services emerged dramatically more than ever before. Neurosurgeons play important roles in diagnosis, treatment and rehabilitation of neuro spine illnesses or injuries (“New generation diseases threaten the community health,” 2008; “Stroke among young people,” 2012). Therefore planning appropriate strategies to retain neurosurgeons in nonmetropolitan or underserved regions of the country has a great importance to properly respond population needs related to these kinds of services (*Medical Workforce In Rural and Remote Australia*, 1996). A real concern is how to maintain these specialists in places where they are most needed. An effective response to this concern depends on identifying factors influencing neurosurgeons’ decision whether to stay or leave their work location after a while. Having a good understanding of these factors is critical because they act as a basis for designing and implementing retention strategies (Chopra, Munro, Lavis, Vist, & Bennett, 2008; *Increasing access to health workers in remote and rural areas through improved retention: Global policy recommendations*, 2010). There is a vast research conducted internationally to identify factors affecting physicians’ decision to retain or leave a work location. Literature addresses some of the contributing factors including satisfying work condition, standard and developed medical equipments and clinical facilities, adequate financial remuneration, opportunities for personal and professional growth, safety concerns, job opportunities for spouse and educational facilities for children (Grobler et al., 2009; Harris, 1999; Hart, Salsberg, Phillips, & Lishner, 2002; J. Humphreys et al., 2009; Lehman, Dieleman, & Martineau, 2008; Scammon, Williams, & Li, 1994; Szafran, Crutcher, & Chaytors, 2001). This study aims to determine the affecting factors from neurosurgeons’ viewpoint to support policy makers in proposing a sort of evidence based retention strategies with the purpose of encouraging neurosurgeons to continue serving in their current work location even those working in remote and underserved areas.

## 2. Methods

A semi structured, qualitative interviews were conducted with 17 in-service neurosurgeons selected from 9 provinces of Iran. We chose qualitative method for this research due to its ability to gain explanatory and in greater detail information about participants’ attitude, opinion and behaviors. Method has also the potential to extend the study results to people with similar characteristics and get a systematic understanding from a definite event or social perspective (*Qualitative Research Methods: A Data Collector’s Field Guide*). This study was approved by Tehran University of Medical Sciences ethics board and agreements were made with each province health deputy with respect to data collection, analysis and reporting the results. Anonymity was guaranteed by masking the names of participants as to ensure the confidentiality of study.

### 2.1 Selection of Surgeons

Seventeen in-service neurosurgeons working in 9 provinces of Iran were chosen to participate in the study. The reason for selecting these nine provinces was to include the attitudes and experiences of surgeons working at different regions of the country (Semnan, Ghazvin, Fars, Kerman, Bushehr, Khoozestan, Sistan Baluchestan, Ghom, Shahrekord) regarding the fact that the attitude of in-service surgeons is varied in places with geographically and epidemiologically diversity. Data collection was completed within 4-5 days in each of the selected communities.

For each province, we included all public hospitals and those health care institutes related to social security organization equipped with neurosurgeon as a specialty. From each hospital, we chose purposefully at least one neurosurgeon willing to participate in the qualitative study. Care was given to ensure representation of both genders, different categories of employment and all geographical locations within the province. Participation was voluntary with no remuneration. Overall, one to three neurosurgeons from each province was participated in the study. Sampling continued until reaching data saturation. By the end of the study, 17 neurosurgeons were interviewed. To be included in the study, surgeons had to be licensed to practice, working in a clinical capacity and retaining in the study province for at least 3 years. We excluded those retired surgeons or those who were sponsored by foreign governments to study medicine.

### 2.2 Data Collection

In-depth, semi structured interviews were conducted to collect data from neurosurgeons about their attitude toward factors influencing retention in current work location even remote and underserved areas. Interview emphasized on avoiding leading questions, inviting explanation of experiences and proper questioning. The researcher conducted individual interviews with each participant by means of an interview guide constructed as a result of a literature review and expert opinions. The interview guide consisted of 7 questions relating to the factors associated with neurosurgeons’ decision to continue serving in a particular work place. Opportunity for further comment was provided in the final question of interview.

Interviews carried out between September and November 2014 and were done either face to face or in some cases by phone lasting about 30 minutes. Each participant was asked the same questions and in the same order of other respondents. All the interviews were conducted and directly transcribed by the same interviewer. Permission to record the interviews was obtained from participants before starting the interview.

### 2.3 Data Analysis

All interviews were transcribed. To increase the validity, the member check strategy was used and comments were incorporated in the final analysis. It helped to ensure that the findings were harmonious with participants’ perceptions, beliefs and opinions. For sorting out the textual data from transcripts, the “framework” approach for qualitative research was used (Ritchie & Spencer 1994). This approach incorporates both inductive and deductive approaches and permits to have an arrangement of pre-determined themes (i.e. Barer et al themes) with those rising from reading the research data (Barer, Wood, & Schneider, 1999). Using a thematic analysis approach, two researchers independently read the transcripts in order to identify key themes (Green & Thorogood, 2009). Thereafter codes were combined and recombined into subthemes. Through this process of study, a coding template incorporated and built upon the framework approach. To improve reliability of the analysis, the software program MAXQDA was used to support the coding of the interview material. Separate code list of each province was compared to create a final code list used for coding all interviews. Member check strategy was used to increase the validity of the study; wherein two researchers used the same qualitative method to analyze the study results, subsequently the findings were compared to ensure the compatibility (Guion, Diehl, & McDonald, 2002).

## 3. Results

A total of 17 neurosurgeons participated in the interview of our qualitative study ranging from one to three interviewees per province. The majority of participants were male (97%), ranging in age from 29 to 56 years old with a mean age of 42.5. Of 17 interviewed surgeons, 16 were married.

All participants were asked about the factors which encouraged them to retain in a particular work location. The corresponding responses were categorized in seven main themes in order of emphasize (Table 1).

**Table 1:** Main and Sub themes of the interview

Main Themes	Sub Themes
Financial/economic factors	Income Loan allowances Retention grants Insurance plans

Professional practice factors	Scope of practice Workload Managerial support Working condition

Clinical infrastructure, equipment and medical supplies	Clinical Infrastructure Availability of medical equipment and devices Quality of medical equipment and devices Availability of clinical support staff

Personal and family factors	Birth origin Place of study Place of one month obligatory rotation Proximity to family Spouse employment Children’s education Socio cultural background Recognition and appreciation

Living condition and comfort	Housing Distance to capital city Transportation Entertainment facilities Safety Weather conditions

Professional education factors	Learning facilities Educational development activities

Governmental policies and management	Distribution system Work contract Regulations and policies

- Financial/economic factors
- Professional practice factors
- Clinical infrastructure, equipment and medical supplies
- Personal and family factors
- Living condition and comfort
- Professional education factors
- Governmental policies and management

### 3.1 Financial/Economic Factors

A large majority of participants agreed that financial incentives play an important variable in making a decision to remain or leave their work location. They mentioned that income level was the main reason for their retention.

*“Money is an important factor. Where the pay is much better, payment schedules more suitable and taxes less in amount, specialists would be encouraged to retain”*.

*“You know here is an underdeveloped area with many difficulties to live and remuneration not satisfying to make it worth to work. If I were pleased with the amount of money I gained from working here, then I could have put up with difficulties easier”*.

*“Medical university of this area doesn’t pay specialists adequately and on time. It takes about half a year to pay physicians’ fee for services”*.

Some of the participants interpreted financial issue as “ability to make earnings”. They acknowledged that possibility to improve income levels by running a private practice definitely affected their choice to retain in a workplace. Many participants complained that there was no opportunity for private practice any more, the common way for former specialists to increase their governmental salary. Therefore they considered their salary to be unfair and inadequate compared to the effort devoting to become a neurosurgeon. Many of the respondents were displeased with current levels of salaries and mentioned the fact that their income was lower than those of previous colleagues.

*“By 2013, my colleagues were able to work in private hospitals or run their own offices to visit patients besides working in university hospitals. This would bring them additional income. But since then, we have to work in university hospitals for a whole day without the permission to have our offices at the same time. MOHME should consider this loss of income and compensate it for us as a kind of incentive”*.

Participants acknowledged that fee for service was the main form of payment to physicians in many areas which did not serve them well. They believed that fee for service would encourage competition among physicians in attracting more patients. Consequently, in smaller communities physicians often visit a small number of patients and accordingly make a reduced amount of income comparing to large cities with considerable population. This concern would be discouraging for most of the physicians working in small, underserved areas near to large municipalities.

*“I think further on-call reimbursement, raising fee for service amount and assigning a considerable fix payment can significantly balance unfair salaries”*.

Another financial issue mentioned by some of the participants was loan allowances or retention grants given to physicians in some working areas. They stated their willingness to establish an imaging or rehabilitation center in their work location, Therefore public assistance in the form of low interest loans would be beneficial for them to use.

*“Loan allowances to establish an imaging center are not only beneficial for the community but also encouraging for physicians to keep on working in underserved areas”*.

*“If I could have bought a proper house in this area, I might be able to persuade my family to live here with me”*.

### 3.2 Professional Practice Factors

Most of the participants declared that their main concern was to have an opportunity to use all the knowledge and skills related to their specialty in a community. Having a limited scope of practice with limited number of patients was a serious apprehension for physicians in rural or underserved communities.

*“In rural communities, the patient population is not large and diverse enough to support clinical and research activities of many specialists. That would be a great challenge for me as a physician because I’d like to keep up whatever I have learned and not let it forget”*.

*“The major concern is that a large number of patient population travels to metropolitans and large urban cities for treatment. Unfortunately they rarely trust physicians in rural or remote communities”*.

Workload was another subject pointed out by participants. Most of them assumed that although they were eager to have sufficient number of patient population, but heavy burden of workload because of deficiency in the number of specialists in a community led to a major problem.

*“High volume of work makes it impossible for me to pursue new opportunities for updating my knowledge or communicating with other specialists”*.

*“Working alone in an area makes a disaster for regional administrators when requesting a day off to leave the community for any reason”*.

Participants also complained about not having time for vacations, training or illness leave when they were alone to serve in a community.

*“We are required to find a substitute for time absent from practice; if not we were enforced to keep on working”*.

They believed that manageable workloads with rational flexibility would make a reasonable work-life balance for them.

“How can I retain in a community where I’m going to be on call every night?”

Heavy workloads would also result in long waiting lists difficult to manage.

*“It is obvious that long waiting lists as a result of just having one specialist in a community are against to the goal of providing health for all”*.

Another area of concern mentioned by the majority of participants was the fact that administrators of medical universities, hospitals and health care facilities should pay attention to provide an appropriate working condition for physicians. They stated unwillingness to retain in a workplace with unsupportive management, careless about physicians’ needs and provision of work requirements. Those participants who stayed more than three years in their workplace all agreed that support from their relevant university was decisive in their decision to stay.

*“Our main intention is to provide the most appropriate health care possible for people but there is always many obstacles keep us away from doing the right thing. Lack of resources, equipment and other health care professionals are some of the difficulties can be mentioned here”*.

*“I believe that positive and supportive attitude of management can encourage physicians to retain in remote areas”*.

Some participants revealed their eagerness to work in a collaborative working condition. Although they mentioned a degree of autonomy necessary in the workplace, they put emphasize on the importance of teamwork and possibility to share knowledge and experience among specialists both within and between communities.

*“That would be beneficial working together as a group, learning from each other and to share the experiences together”*.

*“Many times I encountered with complicated clinical cases which definitely there were a need for colleagues’ consultation, but no help was available for me due to remoteness and difficult accessibility”*.

### 3.3 Clinical Infrastructure, Equipment and Medical Supplies

Concerns about availability of appropriate clinical infrastructure such as operation theatre facilities, medical equipments, CT scan, MRI, Radiology Centers, essential drugs, laboratories and rehabilitation centers were among influencing factors emphasized by all participants. Most of them complained about the disparity and inequality of clinical facilities in their work place.

*“In my work location there is no MRI or even CT scan; those patients who need more advanced diagnostic procedures are being referred to other cities. In this way I lose a large number of patient populations and this is really dissatisfying. Patients who are referred to metropolitans or large cities for MRI definitely prefer to get treatment from urban physicians believing that urban medical centers provide superior health care”*.

They confirmed that lack of clinical infrastructure would limit their capacity to use their skills and provide good quality services for the community. They argued that physicians were less expected to remain in communities be short of equipment, supplies and other main infrastructure.

*“When essential drugs and surgical devices are not available or low quality versions are in hand, how can I serve patients the best possible?”*.

*“Although I have the appropriate knowledge and skill but I am bounded here with no adequate facilities”*.

*“After implementing polices related to health reform plan, hospitals were obligated to supply drugs and medical devices by their own and people didn’t have to pay any more. Although this was somehow a useful plan for patient population; but after a while hospitals found themselves in a difficult situation to meet the expenses. To manage the difficulty, most hospitals decided to contract with limited number of medical device companies offering low-priced but low quality products. This is really a challenging issue demands a serious concern”*.

Availability of anesthesiologists, nurses and physiotherapists as a supporting team of providers was focused as an important concern by most of the participants. They confirmed that besides having a reasonable level of infrastructure, a good team of health providers could help them to run treatment procedures as good as possible. They added that policies which concentrated on resource accessibility and adequate staffing might play as effective means of improving the possibility of physician retention in their work place.

*“I would not function effectively if I didn’t have help”*.

### 3.4 Personal and Family Factors

Participants believed that birthplace could play an important role in their decision to continue serving in a particular community. They emphasized their willingness to work in their homeland and added that they were always looking for such an opportunity.

*“I believe that assigning physicians to work in their own communities will successfully improve retention rates”*.

*“My maternal home is here and that was the main intention of mine to return and serve my own community after graduation”*.

They also declared that being graduated from a medical university located in a particular community could provide them an opportunity to become familiar and adapted with the area environment. From their point of view experiencing culture and overall condition of the area before being graduated was the main important motive for surgeons’ retention in such communities.

*“Here is the city I was graduated from; people, their culture and health needs, administrators and colleagues are all familiar to me and that’s a great advantage to retain here for work”*.

Participants acknowledged that those who have passed their one-month obligatory rotation in an area were more likely to attract and keep on working in such communities. The reason was positive insight they might have got from the initial contact with the city.

*“When I was selected to serve in this city for one-month obligatory rotation it was a good chance to gain valuable experiences and become familiarized with the community”*.

In fact personal influences were one of the main factors that participants agreed on their impact on decision to retain or leave. The desire to be near family was another main concern mentioned by almost all participants. Most of them declared that opportunity for their spouse employment in the area of their work place was another influencing factor in deciding where to continue their practicing.

*“Here is the place where my wife works and my children go to school. Being near to my family makes me satisfied and feel relieved to keep on practicing”*.

*“I live with my mom and dad. They are around seventy years old dealing with heart disease and high blood pressure. Once I was assigned to work here, I mentioned it as a great opportunity for them to take healthy breaths rest of their life in a community with no air pollution, noise and rowdiness”*.

Education of children and availability of good quality schools was one of the main factors affecting participants’ decision to remain in a community. Most of them believed that urban and developed areas provided better future for children. As a result they preferred to leave rural areas before their children started formal schooling.

*“The main concern is to find a suitable high school for my children; I don’t want to destroy their future because of depriving them from high quality school and qualified teachers”*.

Attracting to the lifestyle or integrating with socio cultural background of the community also being engaged in the community through getting administrative position were other personal factors they pointed out as influencing issues in their decision.

*“I think having a positive outlook on things and fit our needs along with community requirements, culture and values are both appealing and helpful to be tied in with working in rural areas”*.

*“Experiencing new culture and learning how to live with different people is satisfying”*.

A number of specialists believed that recognition, appreciation and being accepted as a community member were mainly related to their retention in work locations.

*“I feel much appreciation in this community. People know my family and me by face and name. They behave us in a respectful manner; they give us gifts, farming products and thankful messages periodically. The way they behave, makes me encouraged to continue serving them”*.

*“People of this community are very sympathetic, helpful and supportive. Some of them are active in fundraising and some participate in voluntary activities. It was a long time that community was empty of CT scan until a charity man funded it for the community”*.

*“Last year I got an administrative post in educational deputy of the university; I am satisfied with job and that is the reason to retain”*.

### 3.5 Living Condition and Comfort

General living conditions in a community was another important concern expressed by many participants. Good housing, availability of electricity, clean water and proper air conditioning were among most important issues requested by physicians. Some participants complained about the untidiness and low quality of accommodation provided by the government and mentioned it not suitable for their family to stay comfortably.

*“We are health providers for population; we ourselves need to live in a healthy housing with good standard of living. But unfortunately residential facilities are problematic and I can’t get my family here”*.

Participants mentioned that long distance between community and the capital city would increase travel time, related costs and danger to die or being injured due to road or air accidents; that’s why most of them mentioned remoteness as a serious drawback for a community.

*“There is no airport or train facility in the community; every time I want to travel outside the capital I have to spend lots of time to get the capital city then passing through other cities”*.

Unproblematic transportation facilities, nearness to an airport and safe road and rail network were also emphasized as influencing factors by participants.

*“Fortunately, there is an airport in the community so I can easily travel”*.

*“Road accident rates are unfortunately very frequent in this region; this fact makes me frightened every time I have to travel by vehicles”*.

Living in remote areas means to be far away from entertainment facilities. Most participants agreed that there were not adequate opportunities for entertainment and leisure pursuit in their work location.

*“I believe that a community with proper transportation facilities, access to shops or entertainment centers and overall providing better life condition can be more successful in attracting professionals from all around the country”*.

*“It’s a long time that I haven’t gone to a cinema to watch a movie. At weekends, my wife and I get really confused about where to spend our leisure time since there are no alternatives available”*.

Another important issue was safety. Some of the participants expressed their experiences of being assaulted by community members because of poor medical practices. They demanded security guards in their workplace to help them in such cases. Some also noted difficulties in living in communities with political violence or domestic conflicts.

*“Unfortunately this area is not a safe place to live. Political insecurity, theft and criminal events are common here”*.

On the other hand, some participants believed that living in a nonmetropolitan area had a great benefit for short distances between different places within the community and little time spent for transportation comparing to urban areas.

*“In urban areas we waste most of our time in transportation; but unlike to urban areas it takes just a few minutes to arrive at work place without getting stuck in terrible frustrating traffics”*.

The last cited issue related to the living facilities of a community was the weather condition.

*“I hate warm and humid weather; unluckily the dominant weather condition in this area is in a similar way”*.

### 3.6 Professional Education Factors

Opportunity for continuing learning and facilities available for updating knowledge in the field of specialty were among factors that participants emphasized their importance in deciding whether to stay or leave a work location. They noted that training opportunities could empower them to achieve the goal of career development and to deal better with their job requirements.

Lack of effective internet connection, deficiency of libraries with up to date scientific resources, poor group discussions or seminars for sharing knowledge and experience between professionals were the main difficulties in this way.

*“Medical science is developing in a high speed. I have to come with it and get new fruits of knowledge. But here there are no facilities for”*.

*“We have to get a defined number of credits for recertification of practice every year, but the challenge is that in many rural and remote areas, such programs are not available and we have to travel to another city; although it is costly and time consuming it also means leaving patients without a health provider”*.

*“There is a library in a hospital with no professional books or journals and difficulty in Wi-Fi access”*.

They also highlighted that heavy workload, insufficient time and cumbersome rules limited them to access professional development courses and to attend workshops or other training seminars. As a result, they asked medical university officials to provide books, journals, internet access and other opportunities for distance learning.

*“Owing to the workload, I can’t go to training workshops; it’s about 8 months I haven’t left here getting stuck to serve an enormous number of patients”*.

Being isolated in a remote area, far from other specialists’ consultation and dispossessed of knowledge sharing were some of the main concerns of participants. Majority of them believed that continuing educational courses, scientific and professional congresses and group discussions in academic meetings could be a solution for physicians who were professionally isolated due to working in long distance communities. One of the potential consequences of such programs was strengthening communication network among physicians.

*“If I keep learning and share my medical experiences with other physicians, it would be a great chance to get their useful suggestions and support when necessary”*.

### 3.7 Governmental Policies and Management

The importance of having a fair system and policy for allocating and distributing specialists among different areas of the country was emphasized by a number of participants. They required clear and comprehensible policies about distribution, duration and place of practice. Absence of such guidelines would make specialists disappointed and feel unfair. As a consequence of such a feeling they would decide to leave work location after a short time.

Participants also demanded a clear contract with transparent policies in which a comprehensive explanation about duration and place of work, wages, benefits and other working conditions was stated. They held that transparency would increase their confidence toward the system and consequently encourage them to continue working in a designated area.

*“My colleague and I had a similar score and qualifications but MOHME designated me to serve in an underserved area and the colleague in a developed metropolitan. They didn’t even explain me the reason. Then, why should I waste my life working in a remote community without any facilities? “*.

Participants also agreed that frustrating regulations and bureaucracies enforced by administrators of medical universities or those accountable in the health system of community obstructed them to work outstanding and be relieved from stressful pressures.

*“When a request to supply essential equipment or establishment a specialized center is confirmed to provide better health services to patient population it takes a long time to put it into practice due to slow pace of administration”*.

*“I have a rural job forces me to live far from my families; therefore management should provide additional leave in order to visit the relatives. But difficulty in taking leave is obvious. To give the permission to leave, I have to find a substitute for myself which is not an easy process. All these obstacles disappoint me from keeping on working in remote areas”*.

Political atmosphere of a community had also an important influence on participants’ decision to stay or leave. Some of them acknowledged that the community they were working in responded to specific events such as health reform policies with delay. They added that poor communication and inconsistency between MOHME and medical universities in some local communities might result in unreceptive perception of physicians toward health reform and its effects on career security, salary and professional development.

*“Once the health reform plan initiated in Iran, I was looking for seeing the results of its implementation in my work place. But comparing to other parts of the country, here is far away from the reform and its consequences”*.

## 4. Discussion

Unfair distribution and retention of physicians is still a worldwide problem. This situation avoids rational delivery of health services to people living in remote or underserved communities facing with limited financial resources and higher burden of diseases (Dussault & Franceschini, 2006; Kirigia et al., 2006; Muula et al., 2006; *Shaping the future*, 2003). This study provides evidence about factors influencing neurosurgeons’ decision to retain in a work location. There are considerable literatures on this issue. Similar to previous studies, findings of this research also revealed a combination of influencing factors including increase in salary, better living conditions, proximity to family, equipped hospitals and health facilities, opportunity for educational improvement and satisfying work environment. Diversity of factors suggested that there should be a combination of financial and nonfinancial incentives to encourage physicians retain in their work location (Alexander & Fraser, 2001; Gillham & Ristevski, 2007; J. Humphreys et al., 2009; *Increasing access to health workers in remote and rural areas through improved retention: global policy recommendations*, 2010; Lagarde & Blaauw, 2009; Lehman et al., 2008; Wibulpolprasert & Pengpaibon, 2003; Williams, D’Amore, & McMeeken, 2007). In this study most cited influencing factors from neurosurgeons’ viewpoint were classified in seven categories of financial factors, professional practice factors, clinical infrastructure and medical facilities of the community, personal and family factors, living condition and comfort, professional education factors, organizational policies and management.

Many of these factors have been confirmed by other national or international studies. In the current study it was found that all participants emphasized the importance of monetary incentives in their decision to retain in a community. Those who haven’t changed their work place for more than three years agreed that a satisfying remuneration system was present. They also confirmed that higher income could compensate the opportunity cost of being away from developed facilities. Almost all of previous studies on retaining neurosurgeons focused on the importance of financial incentives (Agyepong et al., 2004; Crandall, Dwyer, & Duncan, 1990; Gillham & Ristevski, 2007). Hall et al. (2007) found that health workers were eager to move to high income communities with the intention of gaining more monetary incentives (Hall et al., 2007). Literature also acknowledges that loan repayment, direct and indirect financial incentives, higher salaries, allowances and retention grants led to highest rates of service completion and retention among physicians (Barnighausen & Bloom, 2009; Costa, Labuda, McCord, & Gillanders, 1996; Jackson, Shannon, Pathman et al., 2003; Koot & Martineau, 2005; Pathman et al., 2004; Rourke, Incitti, Rourke, & Kennard, 2003; Scammon et al., 1994; Sempowski, Godwin, & Seguin, 2002; Wibulpolprasert & Pengpaibon, 2003; Yang, 2003). Although most studies agreed the influence of monetary incentives on physicians’ retention it was noted that multifaceted approaches had greater success than those solely applying financial incentive programs (Barnighausen & Bloom, 2009; Sempowski et al., 2002; Willis-Shattuck et al., 2008; Wilson et al., 2009).

The second category of factors influencing retention of neurosurgeons were work condition related issues such as workload, reasonable on call schedules, an available locum program, management support, access to specialists and referral networks which have been emphasized by several authors (Awases, Gbary, Nyoni, & Chatora, 2003; Cavanagh & Coffin, 1992; Dieleman, Toonen, Touré, & Martineau, 2006; Dieleman, Viet Cuong, Vu Anh, & Martineau, 2003; Hegney, McCarthy, Rogers-Clark, & Gorman, 2002; Irvine & Evans, 1995; Kunaviktikul et al., 2001; V. A. Lambert & C. E. Lambert, 2001; MacPhee & Scott, 2002; Manahan-Roberge & Lavoie, 2008; Zurn et al., 2010). Unmanageable Workload, lack of leisure time, inflexible working hours, lack of managerial support and professional isolation were mentioned as dissatisfying factors (Barer et al., 1999; Carter, 1987; Dussault & Franceschini, 2006; Holmes & Miller, 1986; Kruger & Tennant, 2005; MacPhee & Scott, 2002; Rourke et al., 2003; Sempowski et al., 2002; Sims, 2003; Yang, 2003). Dussault and Franceschini (2006) acknowledged that workers are less likely to retain in a workplace with unsupportive management, lack of equipment and other important infrastructure (Dussault & Franceschini, 2006). Our study also revealed that a pleasing work environment could be achieved through a broad scope of practice and sufficient number of patients available for physicians. Study findings added that although physicians were satisfied with a degree of professional autonomy but a collaborative working condition would also give them pleasure. Literature advocates findings and mentions that while autonomy, accountability and broad scope of practice have been regarded as encouraging results of rural practice (Alexander & Fraser, 2001; J. Humphreys et al., 2009; Keane, Lincoln, Rolfe, & Smith, 2013; Williams et al., 2007), but lack of teamwork and isolation of physicians in remote areas may limit professional development (Douglas, 1991; Marquez & Keane, 2002).

Availability of clinical infrastructure and medical facilities in a community were the third category of affecting factors mentioned by participants of the study. They focused that lack of hospital resources, clinical infrastructure, MRI and other diagnostic facilities limited their capacity to provide good quality services for community. Literature affirms that availability of clinical infrastructure should be essentially taken in to account otherwise patient care cannot be effectively provided (Agyepong et al., 2004; Awases et al., 2003; Dieleman et al., 2006; Dieleman et al., 2003; J. Humphreys et al., 2009; Kotzee & Couper, 2006; Manongi, Marchant, & Bygbjerg, 2006; Mathauer & Imhoff, 2006; Ramani, Rao, Ryan, Vujicic, & Berman, 2013; Ssengooba et al., 2007). Studies also revealed similar findings and mentioned lack of materials as an important de-motivator for physicians’ retention (Alexander & Fraser, 2001; Barnighausen & Bloom, 2009; Dussault & Franceschini, 2006; Gillham & Ristevski, 2007; J. Humphreys et al., 2009; Jackson et al., 2003; Kruger & Tennant, 2005; Lehman et al., 2008; Lu, White, & Barriball, 2005; Pathman et al., 2004; Richards, 2001; Rourke et al., 2003; Williams et al., 2007). Butterworth et al. (2008) emphasized that to facilitate a good health care system it was needed to have a qualified team of physicians and support staff with reasonable level of infrastructure.

Our study distinguished the forth group of affecting factors categorized as personal and family issues including birthplace, university of study, proximity to family, spouse desires, life style habits, recognition and appreciation (Daniels, Vanleit, Skipper, Sanders, & Rhyne, 2007; Grobler et al., 2009; O’Toole, Schoo, Stagnitti, & Cuss, 2008). Different studies confirmed that geographical origin of health workers contribute to their decision to stay in a rural community (Alexander & Fraser, 2001; Brooks, Walsh, Mardon, Lewis, & Clawson, 2002; de Vries & Reid, 2003; Hall et al., 2007; Nitayarumphong, Srivanichakom, & Pongsupap, 2000; H. K. Rabinowitz, Diamond, Markham, & Paynter, 2001; H. K. Rabinowitz, Diamond, Markham, & Rabinowitz, 2005; Walker, Dewitt, Pallant, & Cunningham, 2012; Wibulpolprasert & Pengpaibon, 2003). Whether students with rural origin would return to underserved areas to practice was notably discussed in the literature (Brooks et al., 2002; Colwill, 2003; de Vries & Reid, 2003; H. K. Rabinowitz et al., 2001; H. K. Rabinowitz et al., 2005; Smith & Thomas, 1998; Walker et al., 2012). There was also considerable agreement in research that rural upbringing increased the chance of health workers returning to practice in rural communities (H. K. Rabinowitz et al., 2001; Woloschuk & Tarrant, 2002). A Canadian review also emphasized on geographic origin, graduated in rural areas and financial remuneration as influencing factors (Audas, Ryan, & Vardy, 2009; Carter, 1987; Chomitz et al., 1998; Duplantie et al., 2007; Mathews, Rourke, & Park, 2006; Rosenblatt, Whitcomb, Cullen, Lishner, & Hart, 1992). In our study participants declared that one month compulsory rotation in rural areas could be beneficial to positively form physicians’ perception about rural practice. Literature affirms the statement and explains that those who had participated in rural rotations as residents, considered themselves more eager and prepared to retain in remote work areas (Barrett, Lipsky, & Lutfiyya, 2011; Brooks et al., 2002; Campos-Outcalt & Senf, 1999; Erney, Allen, & Siska, 1991; Wilson et al., 2009). Similar to our study, a number of reviews identified employment of practitioner’s spouse, eagerness to adopt a rural life style, educational opportunities for children and proximity to family as most influential factors in physician retention (Douglas, 1991; Gillham & Ristevski, 2007; Henry, Edwards, & Crotty, 2009; Sims, 2003). Recognition and appreciation was another issue emphasized in most of the literatures (Alexander & Fraser, 2001; Barnighausen & Bloom, 2009; Dieleman et al., 2006; Dieleman et al., 2003; Dussault & Franceschini, 2006; Gillham & Ristevski, 2007; Hall et al., 2007; Jackson et al., 2003; Kotzee & Couper, 2006; Kruger & Tennant, 2005; Kyaddondo & Whyte, 2003; Lehman et al., 2008; Lu et al., 2005; Manongi et al., 2006; Mathauer & Imhoff, 2006; Pathman et al., 2004; Richards, 2001; Sempowski et al., 2002; Ssengooba et al., 2007; Williams et al., 2007). Health workers in Vietnam mentioned the most encouraging factors in their job including appreciation by managers, colleagues and community (Dieleman et al., 2003). Our study found that physicians were satisfied with positive feedbacks from population giving them a perception of being a useful provider for society by serving patient population in a rural and remote community.

Location specific factors and living facilities also played a significant role in participants’ decision to stay in a workplace. These factors included good quality housing, availability of transportation and entertainment facilities, adequate schools for children, safety, nearness to capital city and weather condition (Lehman et al., 2008). The importance of appropriate living condition, schools with qualified teachers, good quality drinking water, electricity, safe roads and proper transportation was emphasized by several international studies (Cavender & Alban, 1998; J. Humphreys et al., 2009; Kim, 2000; Lehman et al., 2008; Nitayarumphong et al., 2000). Furthermore findings of different studies suggested that rural communities could improve physicians’ retention by promoting their living facilities and entertainment amenities also absorbing functional budgets to be spent for developing transportation facilities, building schools and other educational centers (Stretton & Bolon, 2009; Veitch et al., 1999). Thailand invested heavily in rural infrastructure, staff housing and basic infrastructures such as roads, phones and water supplies (Nitayarumphong et al., 2000). Imperfect educational opportunities and lack of schools for children were cited globally as a main reason for health workers’ leave from remote areas (Cavender & Alban, 1998; Kim, 2000; Koot & Martineau, 2005). Remoteness of community to a capital city was also recognized as a significant drawback especially in the absence of airport or rail network (Garnett et al., 2008; Williams et al., 2007). Local geography might also contribute to physician retention if they are interested in natural environment or pleased with easy commute than in urban areas (Stretton & Bolon, 2009; Williams et al., 2007). The reason was increase in travel times and related expenditures. Many participants also underscored that they were not able to cope with rural lifestyle mainly those who were used to urban life. Literature acknowledged that living in remote or underserved areas was associated with lack of social life and entertainment facilities (Ramani et al., 2013). Political stability and safety was another crucial factor emphasized by many participants. Those working in communities with political violence and domestic conflicts were dissatisfied with insecurity of their work place and mentioned it the major reason to leave (Butterworth & Hayes, 2008). A systematic review study conducted by European Union emphasized the importance of domestic violence as a reason to physicians’ move and change their work location (*Important factors regarding recruit and retain in remote rural areas*, 2013). Another aspect of safety was also emphasized by participants as a great concern. They declared that due to remoteness and less control over underserved settings, there should be a definite solution to improve and maintain safety of physicians in the workplace. They demanded physician support programs to reduce hostility and violence in such places. Literature confirmed our study findings and stated some policies and procedures for coping with aggressive patients (Ellenbecker, Samia, Cushman, & Porell, 2007). Political stability and security of the community was also the main concern of study participants (*Important factors regarding recruit and retain in remote rural areas*, 2013). Butterworth emphasized that the need for political consistency and safety was a major issue particularly for physicians working in rural or remote areas (Butterworth & Hayes, 2008).

Our study highlighted the value of professional development, continuous learning and knowledge update as important factors influencing retention of participants in their workplace. The importance of career improvement and learning environment was consistent with literature findings (Agyepong et al., 2004; Awases et al., 2003; Chomitz et al., 1998; Crandall et al., 1990; Dieleman et al., 2006; Dieleman et al., 2003; Glazebrook & Harrison, 2006; Harding, Whitehead, Aslani, & Chen, 2006; *Increasing access to health workers in remote and rural areas through improved retention: global policy recommendations*, 2010; Kotzee & Couper, 2006; Kyaddondo & Whyte, 2003; Mangham & Hanson, 2008; Manongi et al., 2006; Mathauer & Imhoff, 2006; McLean, 2006; Mills & Millsteed, 2002). Consistent with the literature, study participants mentioned some of the barriers to knowledge improvement. Heavy workload, lack of time, policy and funding support were among mentioned factors (Gillham & Ristevski, 2007; Glazebrook & Harrison, 2006; Townsend, Sheffield, Stadnyk, & Beagan, 2006). Literature suggested library access, tele health and distance learning as a number of strategies to manage the barriers and reduce the sense of professional isolation for underserved and remote health workers (Bynum, Irwin, & Cohen, 2010; Dussault & Franceschini, 2006; Gagnon, Duplantie, Fortin, & Landry, 2007). As many rural physicians are unable to attend conferences and workshops due to workload, long distance and travel costs, Butterworth et al proposed distance learning, provision of libraries with update scientific resources and internet access as a solution. Distance learning and providing appropriate facilities for information communication were supposed to overcome some of the shortcomings related to physicians’ isolation (Duplantie et al., 2007; Wilkinson et al., 2001). Mathauer et al. (2006) also highlighted the importance of continuing education and professional development as an approach to encourage health providers in the public sector.

Political environment was mentioned as the last affecting category of factors by study participants. Mathews et al described poor work condition from the view of relationship with managers and amount of bureaucracies and rigid regulations ruling the work place (Mathews et al., 2006). Ramani et al found that since rural physicians had to live far from their families, they required additional leave to visit the relatives more frequently (Ramani et al., 2013). Difficulty in taking formal leave was one of the reasons for their dissatisfaction with the workplace. Our study confirmed the findings and mentioned a flexible workload, managerial support and compassionate regulation as important role players in helping physicians work outstanding and manage the stressful matters of rural practice.

Another issue highlighted by some of the study participants was the necessity for a fair and accountable system of distribution and transfer within the health system facilities. Owing to lack of clear guidelines or non adherence to certain principles, physicians without political influence feared to be chosen to serve in remote areas. Anxiety about MOHME’s policies was apparent in most interviews. Similar to our study literature agrees that success of retention strategies depends on the supportiveness of a broader system within which they are located (Dovlo, 1999; Reid, 2003). Findings highlighted the need for improved communication with MOHME regarding to transparency in the service contract; remuneration, length of service and other working conditions (Ramani et al., 2013). Participants suggested that defined terms of service contract would provide them confidence and peace of mind (Dieleman et al., 2003; Kruk et al., 2009). Contracts with reliable endpoints were emphasized by most of the participants. Improved communication with hospital administrations and medical university officials was another main concern expressed by study participants. They emphasized on managers’ inspiring role in supporting equitable provision of resources and bringing together a professional team (Keane et al., 2013). Supportive management policies including flexible contracts and work conditions also funding for continuing professional education were recommended in the literature (J. Humphreys et al., 2009).

## 5. Conclusion

Neurosurgeons play important role in diagnosis, treatment and rehabilitation of neurological and related spine illnesses and injuries. Planning appropriate strategies to retain neurosurgeons in nonmetropolitan or underserved regions of the country has a great importance to properly respond population needs related to these kinds of services. For this purpose it is required to identify factors affecting neurosurgeons’ decision to retain in a workplace and accordingly propose evidence based strategies to maximize the retention rate of such a short supply and valued resources (J. Humphreys et al., 2009). Having a good understanding of these factors is critical because they act as a basis for designing and implementing retention strategies. According to neurosurgeons interviewed, most of them decided to stay in their workplace because of financial incentives. Of equal importance, work related factors such as having adequate patient volume, reasonable workload, sufficient clinical facilities and managerial support have been recognized as retention factors. Clinical infrastructure of the community and living facilities were cited as other important role players. Other retention factors related to physicians’ characteristics and personal issues including their birth origin, place of graduation, proximity to family, spouse’ employment and educational environment for children were also important considerations. The study also highlighted the significant effect of political climate, managerial rules and educational opportunities as affecting factors. Considering a range of factors, our findings underline the importance of proposing a multi-faceted and holistic strategy that addresses the range of factors identified in this study to maintain neurosurgeons in places where they are most needed (Buchan, 2010; Buchan & Seccombe, 1991; J. Humphreys et al., 2009; *Increasing access to health workers in remote and rural areas through improved retention: global policy recommendations*, 2010; A plan for physician retention in rural and underserved areas,” 2014; Ramani et al., 2013; Wibulpolprasert & Pengpaibon, 2003; Wilson et al., 2009).

## Footnotes

**Notes** Note 1. (Evaluation of health evolution plan in the field of treatment; A plan for physician retention in rural and underserved areas; A series of instructions for health evolution plan, 2014). Note 2. (A plan for physician retention in rural and underserved areas, 2014; A series of instructions for health evolution plan, 2014).
